# Spillover and quantile linkage between oil price shocks and stock returns: new evidence from G7 countries

**DOI:** 10.1186/s40854-020-00208-y

**Published:** 2020-12-12

**Authors:** Yonghong Jiang, Gengyu Tian, Bin Mo

**Affiliations:** 1 Institute of Finance, Jinan University, Guangzhou, China; 2 Institute of Finance, Guangzhou University, Guangzhou, China

**Keywords:** Oil supply shock, Oil aggregate demand shock, Oil specific demand shock, Stock market, Spillover effect, Quantile-on-quantile, C32, F3, G15, Q4

## Abstract

The link between crude oil price and stock returns of the Group of Seven (G7) countries (Canada, France, Germany, Italy, Japan, the United Kingdom, and the United States) was analyzed in this study using monthly data from January 1999 to March 2020. We adopt a similar approach to Kilian (Am Econ Rev 99(3):1053–1069, 2009) and construct a structural vector autoregression framework to decompose crude oil price shocks into oil supply shock, oil aggregate demand shock, and oil-specific demand shock. We then explore the distinct effects of different kinds of oil price shocks from various sources. Based on the decomposed oil price shocks, we apply the connectedness approach and QQ regression to find time-varying co-movements and tail dependence between oil price shocks and G7 stock returns. There is no general correlation between the decomposed oil prices and stock returns in these countries. The effects of oil price shocks on stock returns across different stock market conditions appear to be heterogeneous. Oil supply shock appears to be a net transmitter of spillover effects for all G7 countries within the sample period.

## Introduction

In recent years, the Organization of the Petroleum Exporting Countries (OPEC) and non-OPEC oil producers have reduced oil production in an effort to drive up oil prices. Since oil is an input factor, an increase in price raises the production costs of enterprises from oil-importing countries. The extension of production costs could reduce the profits of companies and lead to a decline in corporate output. Oil price volatility also significantly affects inflation (Cuñado and De Gracia 2005; Cuñado et al. 2015), which decreases consumption by allowing consumers to cut expenditures in other areas (Narayan and Narayan 2007; Leung 2010).

In addition to its general attributes as a commodity, crude oil can also be viewed as a strategic material. The volatility of oil price and supply are significantly affected by political situations. Recent political multi-polarization and internationalization of the production and control of the crude oil market have created turmoil. Geopolitical tension has strengthened the expectations of the international crude oil market due to the shrinking supply. The COVID-19 outbreak has led to further international financial turbulence, disrupting asset allocations, risk management models, and financial stability across the globe. Oil price fluctuations will continue to undoubtedly have an enormous impact on the economies of many countries.

Within the dividend discount model, the stock price is a discounted value of the company's future net profits; the short- and long-term effects of oil prices on national economies are quickly reflected in their stock markets. In other words, the effects of oil price shocks are immediately reflected in the stock price if the stock market is effective. Stock market volatility, which is caused by many factors, has an extraordinary impact on the price of crude oil. The economic situation affects the demand for oil as the stock market reflects the actual real-time economic environment (Chen 2014). The continuous development of the international financial system, including speculation based on financial instruments (e.g., oil options and futures), makes the financial attributes of crude oil increasingly important. At present, stocks are a major cause of crude oil price fluctuations (Zhang 2013). Further, in terms of the extreme risks of the stock market, a sharp rise in stock prices may indicate that the economy is overheating, leading to a sharp increase in oil demand. Conversely, a steep drop in stock prices often indicates an economic downturn or an increase in economic uncertainty. This, in turn, keeps oil speculation relatively conservative.

There has been a great deal of scholarly interest in the systematic risk affecting crude oil and stock markets.

Previous researchers have not considered the factors of various sources (e.g., supply, demand, specific demand) responsible for crude oil price volatility. When crude oil price or oil volatility is used as the basis for a stock market shock analysis, the feedback reflected in stock prices is uniform regardless of the precise factors causing oil prices to move. This can make the final conclusions inconsistent with the actual effects. In this study, we construct a tree-variate structural vector autoregression (SVAR) model (Kilian 2009) to decompose crude oil price into oil supply shock, oil aggregate demand shock, and oil-specific demand shock before analyzing the relationship between oil prices and stock returns.

A functional understanding of the correlation between oil prices and stock returns necessitates an accurate analysis of extreme tail event risk and its time-varying impact on the market. The goal of this study is to compare the different quantile features and dynamic spillover effects in the correlations between three kinds of decomposed oil price shocks and stock returns in the Group of Seven (G7) member countries: Canada, France, Germany, Italy, Japan, the United Kingdom, and the United States. International oil price fluctuations affect investor behavior in listed companies across related industries, thus affecting stock price index and increasing risk spillover. The rapid development of the G7 economy and the massive global consumption of oil also place G7 in a vital role in the international oil price market. The volatility of G7 stock markets is an important basis for policymakers in other countries as they judge economic trends and make decisions. We apply a quantile-on-quantile (QQ) approach to estimate the tail-dependence performance between decomposed oil price shocks and varying stock return quantiles. This approach allows us to estimate multiple quantiles in both variables and is well-suited to the extreme risk problems at hand. We also capture the direction and scale of time-varying spillovers between decomposed oil price shocks and stock returns via the connectedness index method.

Our study contributes to the related literature in the following aspects: first, previous studies merely focused on either the extreme tail risk between oil and stock markets (Lin and Su 2020), or time-varying characteristics and directional risk contagion (Antonakakis et al. 2017; Nadal et al. 2017). We extend the literature (Bastianin et al. 2016) about the relationship between oil price and G7′s stock returns by covering both aforementioned aspects and identifying the different oil shocks using the SVAR method proposed by Kilian (2009). Second, relative to some early research, we use some novel methods, that is, the time-varying parameter vector autoregression (TVP-VAR) and QQ models. Based on the connectedness framework (Diebold and Yilmaz 2012), the TVP-VAR approach can dig into the dynamic directional spillover risk between different oil price changes and market conditions (Antonakakis et al. 2017). The modified TVP-VAR does not lose observations when utilizing a fixed window size during the empirical process, making our findings more reliable (Antonakakis and Gabauer 2017). Compared with the routine quantile regression model, the QQ method can explore the tail dependence structures in common market conditions (middle quantiles), bullish market conditions (higher quantiles), and bearish market conditions (lower quantiles) (Chang et al. 2020). This will make our results dynamic and detailed. Third, our findings are also of essential practical significance. On the one hand, it conducts intensive research into the effect of various oil price changes on stock markets while employing data that takes into account the COVID-19 pandemic. This study further draws similarities and differences between the risk contagion in 2020 and 2008. On the other hand, we put forward several suggestions for policymakers that target the current risks. The tail risk between oil and stock markets has also been taken into account, which helps in constructing and switching to a better portfolio to avoid risks.

The structure of the paper is organized as follows. The literature review is presented in “Literature review” section. The dataset and methodology are indicated in “Data and methodology” section. The empirical results are illustrated in “Empirical results” section, and the conclusions and political suggestions are covered in “Conclusion” section.

## Literature review

Many researchers have investigated the effects of oil price shocks on macro-economic activities. Hamilton (1983) was the first to discover that oil prices caused an economic recession after World War II. Mork (1989) expounded upon Hamilton’s (1983) work to find that the US gross domestic product (GDP) is affected by oil price volatility. Balassa (1985) found that an oil price shock not only contributes to economic growth, but also to exports and policy choices in developing countries. Lee et al. (1995) argued that the impact of oil price fluctuations is more significant in an economy where oil prices are stable than in one where they are fluctuating. Cuñado and de Gracia (2003) found that the European industrial production indexes (IPI) growth rate is asymmetrically affected by short-term oil price volatility. Through another investigation, Cuñado and Gracia (2005) also discovered that the impact of oil prices on Asian economies is more intense when oil is settled in the local currency. Hamilton (2008) conversely claimed that oil price shocks have continuously affected core inflation in the United States since the 1980s.

Du et al. (2010) used a vector autoregression (VAR) model to show that China’s economic growth is positively linked to global oil prices. Morana (2013) found that macro-economic shocks (e.g., liquidity and inventory) are positively correlated with real oil prices. Taghizadeh-Hesary et al. (2016) found that emerging countries are more vulnerable to oil price movement than developed countries. Oladosu et al. (2018) revisited the correlation between macro-variables and oil prices, where the feedback of macro-activities to oil price shocks appear to have weakened since the 1970s. Phan et al. (2019) demonstrated that West Texas Intermediate (WTI) volatility causes a decline in investment expenditures.

The stock market is an essential component of the macro-economy. It is important to understand the manner in which oil price shocks trigger market volatility. Many previous researchers have explored the impact of oil price volatility on the stock market (e.g., Kaul and Seyhun 1990; Jones and Kaul 1996), inspiring others to investigate the effects of oil shocks on stock returns. Oil prices have disparate effects on stock returns in different countries or regions. For example, Sadorsky (1999) applied the VAR approach and found that both oil price shocks and oil price volatility have a crucial impact on US stock returns. Oil price volatility also has an asymmetrical effect on stock returns; Papapetrou (2001) showed that stock returns can be depressed by positive oil price shocks. Park and Ratti (2008) demonstrated that while stock returns in most countries decrease significantly within the same month when oil prices move, the Norwegian stock market shows a uniquely positive correlation with oil price. They found no evidence that the stock returns of oil-importing countries in Europe respond asymmetrically to oil price volatility. Bjørnland (2009) confirmed that stocks rose by 2.5% for every 10% increase in oil prices in Norway, a developing oil-export country.

There have been many other valuable contributions to the literature. Smyth and Narayan (2018) reviewed oil price-stock returns as a popular research subject. Filis et al. (2011) used the Dynamic Conditional Correlation Glosten Jagannathan Runkle Generalized Autoregressive Conditional Heteroscedasticity (DCC-GJR-GARCH) method to find that the relationship between stock markets and oil prices is time-varying. The direction of this impact in both oil-importing and oil-exporting countries shifted during the global financial crisis. Basher et al. (2012) investigated emerging market stock prices to find that the positive impact of oil price tends to lower stock returns. Kang and Ratti (2013) found that stock returns are influenced by oil prices and economic policy uncertainty. Reboredo and Rivera-Castro (2014) used a wavelet multi-resolution analysis to find that, interestingly, oil price movements did not affect US stock prices during the financial crisis.

We drew upon the works of these researchers in conducting the present study as our approach relates to overarching concepts in the literature. The first of these concepts centers on the decomposition of historical oil prices (Kilian 2009), for instance, where Kilian and Park (2009) found that stock prices respond differently to different oil-source shocks. Wang et al. (2013) further analyzed the characteristics of oil exporting and importing countries on the basis of oil decomposition. In a later study, Wang et al. (2014) also decomposed the crude oil price by driving factors and observed the effects of its volatility on agricultural product markets; before and after the financial crisis, agricultural product prices responded distinctly to different sources of crude oil price shocks. Fang and You (2014) studied the relationship between crude oil price shocks and large newly industrialized economies (NIEs); different price-driven oil price movements were shown to affect NIE stock prices differently. There is a partial integration between NIE stock markets and crude oil price shocks.

Cuñado et al. (2015) explored the effects of structured oil prices on mainly Asian oil consumption economies to find that various macro-activities respond heterogeneously to different oil prices. Ji et al. (2015) studied the impact of crude oil prices on the macro-economic activities of BRICS countries, using a SVAR model to decompose crude oil prices according to driving factors. With the exception of Russia, the impact of aggregate demand shocks dominates the BRICS. Ahmadi et al. (2016) analyzed the correlation between crude oil price and the US stock market in various industries by decomposing oil price volatility into specific demand shock, demand shock, and supply shock. The response stock returns to the impact of oil prices appeared to be shock-dependent; demand shock was the most relevant driver of stock returns in their analysis.

Bastianin et al. (2016) found that oil supply shock does not affect the stock market volatility of G7 countries while oil demand shock does. Li et al. (2017) used a SVAR model to decompose crude oil prices into four types of oil price shocks affecting the stock returns of listed companies in China's oil industry chain. The listed companies in this chain showed a significant positive correlation with oil supply shock and precautionary demand shock. Gong and Lin (2018) explored the impact of oil supply shock and demand shock on China's stock returns to find that they were time-varying within the sample range.

The second overarching concept is related to the spillover between oil price and stock returns. Understanding the spillover effect reveals the direction of shocks when volatilities occur, allowing for a sound analysis of the time-varying relationship between oil shocks and stock returns. Based on implied volatility, Maghyereh et al. (2016) found that many sectors are subject to the net spillover effect of oil on equity markets. Diebold et al. (2017) studied the spillover effects between different categories of commodities and found that the energy sector most significantly impacts the others. Antonakakis et al. (2017) observed spillover effects across decomposed oil price shocks in oil-importing and exporting economies. They found that the total demand shock is the net transmitter among all oil shocks. Zhang (2017) asserted that oil has little impact on stock returns, while crude oil markets acquire significant spillover from international financial markets. Ferrer et al. (2018) and Antonakakis et al. (2018) found that the spillover effects of oil volatility and oil and gas company stocks indeed affect crude oil price fluctuations. Ji et al. (2018) learned that the WTI and its refinery products dominate the correlations between the gas future markets of the United States and the United Kingdom. Husain et al. (2019) demonstrated that stock index volatility considerably impacts commodities, with the greatest spillover effect on oil.

Since the outbreak of the subprime mortgage crisis in 2008, many scholars have conducted in-depth research on financial risk estimation (Kou et al. 2014; Chao et al. 2020; Wang et al. 2020). The majority of research on the correlations between oil prices and stock markets has been based on the VAR approach. Many have explored the impact of oil price volatility under extreme stock market conditions using CoVaR and Copula methods (Aloui et al. 2013; Reboredo 2015; Jiang et al. 2018). Others have used the quantile regression (QR) method to find that extreme stock market performance intensifies the impact of oil on stock prices (e.g., Lee and Zeng 2011; Mensi et al. 2014; Reboredo and Ugolini 2016; Zhu et al. 2016). The effects in different quantiles of both variables can be observed in the QQ frame (Sim and Zhou 2015) based on the QR. The impact of oil price shocks on US stock returns, for example, was detected at bilateral quantiles. In another study, Shahbaz et al. (2018) found that energy consumption is positively related to economic growth. Sharif et al. (2019), Chang et al. (2020), and Jiang et al. (2020) have since used the QQ method to explore globalization, stock markets, and oil. Lin and Su (2020) applied the QQ method to investigate the relationship between oil market uncertainty and stock markets; overall, negative effects were observed in most sample countries, especially during the depression of the Islamic stock market.

The primary mechanism discussed in this paper is closely related to the one presented by Bouoiyour and Selmi (2016). Unlike Bouoiyour and Selmi (2016), however, who used raw Chinese oil price data, we applied the WTI world crude oil to estimate the impact of fluctuations in this study. Though China is the second-largest economy in the world, its oil futures market was only recently established in March 2018 and its oil trading system is not advanced. Additionally, the impact of world crude oil prices on G7 stock returns is more significant (Diaz et al. 2016). We also constructed a SVAR model to decompose crude oil price. Given the dynamic nature of oil prices and stock returns in today’s market, we employed the connectedness spillover method to detect the time-varying interrelationship of decomposed oil prices and stock returns. Finally, we conducted a QQ regression to obtain sufficiently diverse and intuitive conclusions based on different types of oil shock.

There is no scholarly consensus regarding the relationship between oil shocks and stock markets. Most researchers focused on unilateral shock, that is, the impact of oil on the stock market. Unlike previous empirical studies, we aim to systematically analyze the time-varying relationships affecting oil price-stock returns to observe the extreme risks when the stock market or oil shock is dominant. We also explore the dynamic relationships between decomposed oil prices and stock returns. We discuss the co-movement between the different effects of quantiles of decomposed oil prices and those of G7′s stock prices with focus on the asymmetry of tail dependence.

## Data and methodology

### Data

Our study is based on monthly returns data from the stock markets of the G7 member countries (S&P TSX in Canada, CAC 40 in France, DAX 30 in Germany, FTSE MIB in Italy, NIKKEI 225 in Japan, FTSE 100 in the U.K. and S&P 500 in the U.S) for the period covering January 1999 to March 2020.^1^ We compute the stock returns of G7 countries with $SR_{t} = ln(sp_{t} ) - ln(sp_{t - 1} )$, where $sp_{t}$ is the stock closing price of a certain country at period $t$. We focus on G7 countries because they have the most developed economies in the world, accounting for more than 64% of the global net worth and 46% of the GDP. Meanwhile, their economic systems show significant differences in policy interventions, economic reforms, and financial regulation activities. Second, G7 stock markets have become relatively efficient after a long period of development. The impact of oil price shock on the stock market can be more accurately and quickly reflected in stock prices, making the study of the feedback of stock markets to oil price shocks more comprehensive. We obtain the global economic activity index from Kilian's homepage (https://www-personal.umich.edu/~lkilian/). The real oil price is obtained through the nominal price of oil deflated by the US consumer price index (CPI). The growth of world oil production and a normal crude oil price of the WTI are obtained from the Energy Information Administration (EIA). The descriptive statistics of the data are presented in Table 1.

**Table 1:** Descriptive statistics of oil price shocks and stock returns

	Mean	Median	Min	Max	SD	Skewness	Kurtosis	JB	ADF
Supply shock	− 0.001	0.018	− 2.684	2.713	0.848	− 0.008	3.905	8.662**	− 15.894***
Aggregate demand shock	0.028	0.034	− 4.778	3.072	1.052	− 0.314	5.024	38.717***	− 15.719***
Specific demand shock	0.048	0.127	− 5.181	2.276	0.955	− 0.855	5.819	70.946***	− 15.264***
CAC 40	0.000	0.008	− 0.192	0.126	0.052	− 0.691	4.116	33.379***	− 13.914***
DAX 30	0.003	0.075	− 1.019	0.464	0.275	− 1.510	5.367	155.825***	− 14.115***
FTSE MIB	− 0.000	0.007	− 0.149	0.083	0.040	− 0.792	4.089	39.101***	− 15.067***
N 225	− 0.002	0.002	− 0.222	0.341	0.070	0.224	5.621	74.849***	− 18.048***
FTSE 100	0.001	0.008	− 0.272	0.121	0.056	− 0.771	4.479	48.306***	− 13.795***
S&P500	0.003	0.009	− 0.186	0.102	0.043	− 0.802	4.445	49.346***	− 14.235***
TSX60	0.003	0.008	− 0.179	0.112	0.042	− 1.071	6.144	153.192***	− 12.557***

***, **, *Significance at the 1%, 5%, 10% level, respectively

### Empirical methodology

Our empirical analysis consists of the following three steps. In the first step, we decompose the WTI price and further calculate the decomposed oil price shocks to assess the impact of different kinds of oil price shocks on stock returns. In the second step, we estimate the time-varying linkages between decomposed oil prices and G7 stock returns. Doing this allows the dynamic relationships and transmission mechanism between the aforementioned series to be clearly captured. In the third step, we focus on interrelationships between the decomposed oil prices and G7 stock returns at multiple quantiles, especially at the extreme quantiles.

#### Historical decomposition of real oil price

The approach of decomposed oil price in this study is synthetically used according to the procedure of Kilian (2009). In Eq. (1), the tree-variate SVAR method is constructed to decompose the real oil price:

1 $$A_{0} y_{t} = \alpha + \sum\nolimits_{i = 1}^{24} {A_{i} y_{t - 1} } + \varepsilon_{t}$$

where $y_{t} = (s_{t} ,g_{t} ,p_{t} )^{\prime}$, $\varepsilon_{t} = (\varepsilon_{t}^{SS} ,\varepsilon_{t}^{DS} ,\varepsilon_{t}^{OS} )^{\prime}$, $s_{t}$ is the crude oil supply in log-difference term; $g_{t}$ is the real economic activity index; and $p_{t} = 100ln\left( {\frac{{np_{t} }}{{CPI_{t} {/}100}}} \right)$ is the logarithmical real oil price, where $np_{t}$ stands for the nominal prices of oil and $CPI_{t}$ is the consumer price index.

As shown in Eq. (2), the reduced-form VAR model can be described as:

2 $$y_{t} = \beta + \sum\nolimits_{i = 1}^{24} {B_{i} y_{t - 1} } + e_{t}$$

where $e_{t} = A_{0}^{ - 1} \varepsilon_{t}$ in Eq. (3) is supposed as:

3 $$e_{t} = \left[ {\begin{array}{*{20}c} {e_{t}^{s} } \\ {e_{t}^{g} } \\ {e_{t}^{p} } \\ \end{array} } \right] = \left[ {\begin{array}{*{20}c} {a_{11} } & {0} & {0} \\ {a_{21} } & {a_{{2{2}}} } & {0} \\ {a_{31} } & {a_{{{32}}} } & {a_{33} } \\ \end{array} } \right]\left[ {\begin{array}{*{20}c} {\varepsilon_{t}^{SS} } \\ {\varepsilon_{t}^{DS} } \\ {\varepsilon_{t}^{OS} } \\ \end{array} } \right]$$

where $\varepsilon_{t}^{SS}$ represents the unpredictable innovation in global oil production, which is called crude oil supply shock. $\varepsilon_{t}^{DS}$ represents the innovations in global real economic activity, which stands for oil aggregate demand shock. $\varepsilon_{t}^{OS}$, which means innovation in real oil price, is described as oil specific demand shock.

To get insights into the real oil price shock, the $\hat{\beta }$, $\hat{B}_{i}$, and $\hat{e}_{t}$ are obtained using the VAR model. The orthogonal impulse response functions $I_{q} = \frac{{\partial p_{t} }}{{\partial \varepsilon_{t} }} = \left( {\frac{{\partial p_{t + q} }}{{\partial \varepsilon_{t}^{SS} }},\frac{{\partial p_{t + q} }}{{\partial \varepsilon_{t}^{DS} }},\frac{{\partial p_{t + q} }}{{\partial \varepsilon_{t}^{OS} }}} \right)^{^{\prime}}$, $q = 0,1,2, \ldots$, and the structural shock $\hat{\varepsilon }_{t} = (\hat{\varepsilon }_{t}^{SS} ,\hat{\varepsilon }_{t}^{DS} ,\hat{\varepsilon }_{t}^{OS} )^{\prime}$ are then calculated. In Eq. (4), the Cholesky decomposition based method is utilized to calculate oil supply shock $p_{t}^{SS}$, oil aggregate demand shock $p_{t}^{DS}$, and oil-specific demand shock $p_{t}^{OS}$:

4 $$p_{t}^{SS} = \sum\nolimits_{q = 0}^{t - 1} {\frac{{\partial p_{t} }}{{\partial \varepsilon_{t - q}^{SS} }}\hat{\varepsilon }_{t - q}^{SS} } ,p_{t}^{SS} = \sum\nolimits_{q = 0}^{t - 1} {\frac{{\partial p_{t} }}{{\partial \varepsilon_{t - q}^{DS} }}\hat{\varepsilon }_{t - q}^{DS} } ,p_{t}^{OS} = \sum\nolimits_{q = 0}^{t - 1} {\frac{{\partial p_{t} }}{{\partial \varepsilon_{t - q}^{OS} }}\hat{\varepsilon }_{t - q}^{OS} }$$

where $p_{t}^{SS}$, $p_{t}^{DS}$, and $p_{t}^{OS}$ constitute the real oil price and $p_{t} = c + p_{t}^{SS} + p_{t}^{DS} + p_{t}^{OS}$ with a constant $c$.

#### Volatility spillover index

What we know about spillover index is largely based on the original study of Diebold and Yilmaz (2009). To study the transmission mechanism in a time-varying vision and test the spillover effect of the decomposed oil price shocks and the G7 stock returns, we apply the TVP-VAR connectedness approach proposed by Antonakakis and Gabauer (2017). Unlike the connectedness framework of Diebold and Yilmaz (2012), which needs to arbitrarily set the rolling window size to acquire observations, this TVP-VAR approach provides the convenience of setting the rolling window at once. The framework of the TVP-VAR approach can be expressed in Eqs. (5) and (6) as:

5 $$Y_{t} = \alpha_{t} X_{t - 1} + \varepsilon_{t} ,\quad \varepsilon_{t} \sim N(0,\sigma_{t}^{2} )$$

6 $$vec(\alpha_{t} ) = vec(\alpha_{t - 1} ) + u_{t} ,\quad u_{t} \sim N(0,\sum )$$

where $Y_{t}$ is an $N \times {1}$ dimensional vector and $X_{t - 1}$ is a $P \times 1$ dimensional vector. $\alpha_{t}$ is an $N \times P$ dimensional time-varying coefficient matrix. $vec(\alpha_{t} )$, $vec(\alpha_{t - 1} )$, and $u_{t}$ are $P^{2} \times 1$ dimensional vectors. $\varepsilon t$ is an $N \times {1}$ dimensional error-disturbance vector with an $N \times N$ time-varying variance–covariance matrix, $\sigma_{t}^{2}$. $\sum$ is a $P^{2} \times P^{2}$ dimensional matrix.

According to Koop et al. (1996) and Pesaran and Shin (1998), we express the generalized impulse response function (GIRF) and generalized forecast error variance decomposition (GFEVD) in Eqs. (7) and (8), respectively:

7 $$Yt = \sum\nolimits_{j = 0}^{\infty } {L^{{\prime }} } W_{t}^{j} L\varepsilon_{t - j}$$

8 $$Yt = \sum\nolimits_{j = 0}^{\infty } {A_{it} \varepsilon_{t - j} }$$

where $L = \left[ {M_{N} , \ldots ,K_{p} } \right]^{{\prime }}$ is a $P \times N$ dimensional matrix and $W = \left[ {\alpha_{t} M_{P - 1} , \ldots ,K_{(P - 1) \times N} } \right]^{{\prime }}$ is a $P \times P$ dimensional matrix. We measure the difference on whether variable $i$ is affected by the impact at H-step ahead forecast using Eqs. (9), (10), and (11):

9 $$GIRF_{t} = E\left( {Z_{t + H} |\varepsilon_{j,t} = \delta_{j,t} ,F_{t - 1} } \right) - E\left( {Z_{t + H} |F_{t - 1} } \right)$$

10 $$\psi_{j,t}^{g} (H) = \frac{{A_{H} \sigma_{t}^{2} \varepsilon_{j,t} }}{{\sqrt {\sigma_{jj,t}^{2} } }}\frac{{\delta_{j,t} }}{{\sqrt {\sigma_{jj,t}^{2} } }},\delta_{j,t} = \sqrt {\sigma_{jj,t}^{2} }$$

11 $$\psi_{j,t}^{g} (H) = \sigma_{jj,t}^{ - 1} A_{H,t} \sigma_{t}^{2} \varepsilon_{j,t}$$

where the GIRFs of variable $j$ are represented by $\psi_{j,t}^{g} (H)$; $H$ is the forecast horizon; $\delta_{j,t}$ is a selection vector that is equal to one on the $H$th position, and zero otherwise; and $F_{t - 1}$ is the information of period $t - 1$. The GFEVD, which can be transformed as the variance share variable $i$, explains the variable $j$ through Eq. (12):

12 $$\tilde{\theta }_{ij,t}^{g} (H) = \frac{{\sum\nolimits_{t = 1}^{H - 1} {\psi_{ij,t}^{2,g} (H)} }}{{\sum\nolimits_{j = 1}^{N} {\sum\nolimits_{t = 1}^{H - 1} {\psi_{ij,t}^{2,g} (H)} } }}$$

with $$\sum_{j = 1}^{N} \tilde{\theta }_{ij,t}^{g} (H) = 1,$$$$\sum_{i,j = 1}^{N} \tilde{\theta }_{ij,t}^{N} (H) = N$$. Following Diebold and Yilmaz (2009), there are two kinds of spillovers in this method: variable $$j$$ shocks that affect the error variance of variable $$i$$ at H-step ahead forecast (with contribution $$\psi_{ij,t}^{2,g} (H)$$), and variable $$i$$ shocks that affect the error variance of variable $$j$$ at H-step ahead forecast (with contribution $$\psi_{ji,t}^{2,g} (H)$$).

The direction of the spillover can be detected by the connectedness approach. In Eq. (13), the total connectedness index (TCI) according to the GFEVD is expressed as:

13 $$C_{t}^{g} (H) = \frac{{\sum_{i,j = 1,i \ne j}^{N} \tilde{\theta }_{ij,t}^{g} (H)}}{{\sum_{i,j = 1}^{N} \tilde{\theta }_{ij,t}^{g} (H)}} \times 100 = \frac{{\sum_{i,j = 1,i \ne j}^{N} \tilde{\theta }_{ij,t}^{g} (H)}}{N} \times 100$$

Furthermore, Eq. (14) shows the formula for the spillovers, which variable $i$ emits to all other variables $j$. And are estimated by the total directional connectedness to others:

14 $$C_{i \to j,t}^{g} (H) = \frac{{\sum_{j = 1,i \ne j}^{N} \tilde{\theta }_{ji,t}^{g} (H)}}{{\sum_{j = 1}^{N} \tilde{\theta }_{ji,t}^{g} (H)}} \times 100$$

Next, we use the total directional connectedness shown in Eq. (15) to calculate the spillover effect variable $i$ receives from all other variables $j$.

15 $$C_{i \leftarrow j,t}^{g} (H) = \frac{{\sum_{j = 1,i \ne j}^{N} \tilde{\theta }_{ji,t}^{g} (H)}}{{\sum_{i = 1}^{N} \tilde{\theta }_{ji,t}^{g} (H)}} \times 100$$

The net total directional connectedness index of variable $i$ can then be expressed in Eq. (16) as:

16 $$C_{i,t}^{g} (H) = C_{i \to j,t}^{g} (H) - C_{i \leftarrow j,t}^{g} (H)$$

Finally, Eq. (17) shows the calculation for the net pairwise directional connectedness index (NPDC) between variables $i$ and $j$:

17 $$NPDCij(H) = \frac{{\tilde{\theta }_{ji,t}^{g} (H) - \tilde{\theta }_{ij,t}^{g} (H)}}{N} \times 100$$

Equation (17) illustrates a summary of how much the spillover effect of each variable $i$ contributes to the spillover effect of other variables.

#### Quantile-on-quantile regressions

The third empirical sector of this study is based on the QR method suggested by Koenker and Bassett (1978). Given that the QR method may not show the full and accurate impact of oil price shocks on stock returns, this study’s application of the QQ method is more accurate and exhaustive in the correlation of covariates on the dependent variable.

A QQ regression provides more detailed and far-reaching results than the traditional quantile regression analysis (QRA) method. Moreover, the QRA may overlook the nature of uncertainty, which affects the interaction of correlation. Equation (18) shows the first equation:

18 $$SR_{t} = \beta^{\theta } (Oil_{t} ) + \mu_{t}^{\theta }$$

where $SR_{t}$ represents the stock returns of one economy at period $t$; $Oil_{t}$ is the oil price shock at period $t$; $\theta$ represents the $\theta$th quantile; and $\mu_{t}^{\theta }$ the quantile residue.

The problem of an asymmetric effect that exists at the extreme quantile of oil price shock on the extreme quantiles of G7 stock returns, which the QR may ignore, can be resolved by following Ma and Koenker (2006) and Sim and Zhou (2015). We construct QQ regressions to assess the quantile links between oil price shocks and G7 stock returns. Therefore, the first order Taylor expansion is applied to expand the $\beta^{\theta } ( \cdot )$, which is expressed Eq. (19):

19 $$\beta^{\theta } (Oil_{t} ) \approx \beta^{\theta } (Oil^{\tau } )(Oil_{t} - Oil^{\tau } )$$

where $\beta^{\theta } (Oil_{t} )$ represents the partial derivative of $\beta^{\theta } (Oil_{t} )$ with regard to Oil. $\beta^{\theta } (Oil^{\tau } )$ and $\beta^{\theta ^{\prime}} (Oil^{\tau } )$ represent the parameter function of $\theta$ and $\tau$, so that $\beta^{\theta } (Oil^{\tau } )$ and $\beta^{\theta ^{\prime}} (Oil^{\tau } )$ can be replaced as $\beta_{0} (\theta ,\tau )$ and $\beta_{1} (\theta ,\tau )$, respectively. Equation (19) can then be re-written as Eq. (20):

20 $$\beta^{\theta } (Oil_{t} ) \approx \beta_{0} (\theta ,\tau ) + \beta_{1} (\theta ,\tau )(Oil_{t} - Oil^{\tau } )$$

We can then derive Eq. (21) from Eqs. (18) and (20):

21 $$SR_{t} = \underline{{\beta_{0} (\theta ,\tau ) + \beta_{1} (\theta ,\tau )(Oil_{t} - Oil^{\tau } )}} + \mu_{t}^{\theta }$$

The forepart of Eq. (21) is the $\theta$th quantile of stock returns, which is the response of the diverse impacts of the $\tau$ th quantiles of oil price shocks on the $\theta$ th quantiles of stock returns in G7 economies.

We then use the estimations of $Oil_{t}$ and $Oil^{\tau }$ to replace the original, and the local linear regression’s estimates $b_{{0}}$ and $b_{1}$ could be utilized to replace $\beta_{0}$ and $\beta_{{1}}$. Therefore, Eq. (21) can be calculated by Eq. (22):

22 $$\min_{{b_{0} ,b_{1} }} \sum\nolimits_{i}^{n} {\rho_{\theta } \left[ {EG_{t} - b_{0} - b_{1} (O\hat{i}l_{t} - O\hat{i}l^{\tau } )K\left( {\frac{{F_{n} (O\hat{i}l_{t} - \tau )}}{h}} \right)} \right]}$$

where $\rho_{\theta } (u)$ represents the quantile loss function expressed as $\rho_{\theta } (u) = u(\theta - I(u < 0))$. $K( \cdot )$ is the kernel function, and the Gaussian kernel is applied to weight the results in the neighborhood of $Oil^{\tau }$. Moreover, these weights are reversely related to the distanced observations in $O\hat{i}l_{t}$, expressed as $F_{n} (O\hat{i}l_{t} ) = \frac{1}{n}\sum\nolimits_{k = 1}^{n} {I(O\hat{i}l_{k} < } O\hat{i}l_{t} )$, where $I$ represents a usual indicator function. In addition, $O\hat{i}l_{t}$ is also related to the quantile $Oil^{\tau }$ reported by $\tau$.

To measure the different frequencies of oil price shocks and stock returns more specifically, the bandwidth parameter $h = 0.05$ is considered to weight the observations in the neighborhood of the quantiles (see Sim and Zhou 2015; Shahbaz et al. 2018).

## Empirical results

In this section, we discuss the dynamic time-varying spillover between decomposed oil price shocks and G7 stock returns. We also examine the extreme performance and tail dependence of oil price shocks on stock returns at various distributional levels.

### Dynamic time-varying spillover

We compute the connectedness indices of correlations between the three decomposed oil shocks and stock returns of G7 economies. Tables 2, 3 and 4 report the dynamic connectedness results for volatility spillover values based on full sample estimations. The total volatility spillovers of the three kinds of oil shocks are all above 55%. The spillover of oil-specific demand shock is the highest (56.38%), indicating non-negligible interconnectedness and interdependence among spillover values. Net spillover indices were the focus of this study. We observe that the FTSE100, CAC40, and S&P500 dominate the spillover among the three types of oil shocks; that is, they all have positive spillover effects on oil shocks. The DAX30, FTSE MIB, and N225 are the net receivers of spillover from oil shocks. Oil supply shock is generally impacted by G7 stock returns (− 7.466%), while the impact of G7 stock returns on oil aggregate demand shock is more intense (− 12.711%). Likewise, the impact of specific oil demand is markedly affected by G7 stock returns (− 16.410%). Remarkably, the FTSE100 and FTSE MIB have emerged as the most obvious transmitter and receiver of all three shocks, respectively.

**Table 2:** Dynamic connectedness of oil supply shock

	Supply Shock	FTSE100	DAX30	CAC40	TSX60	FTSE MIB	S&P500	N225	FROM
Supply shock	88.728	2.176	1.566	1.805	2.103	1.47	1.217	0.934	11.272
FTSE100	0.346	26.294	0.507	18.209	15.455	9.186	17.274	12.729	73.706
DAX30	1.451	2.082	86.556	1.951	3.357	1.257	2.204	1.142	13.444
CAC40	0.49	18.957	0.494	27.319	10.931	8.536	19.379	13.894	72.681
S&P TSX	0.533	20.194	0.34	13.525	35.074	7.521	12.774	10.039	64.926
FTSE MIB	0.421	13.578	0.421	12.221	9.443	37.58	14.468	11.868	62.42
SPY	0.292	17.575	0.278	18.992	10.009	10.356	26.626	15.873	73.374
N225	0.274	15.662	0.26	15.811	10.325	9.476	18.183	30.01	69.99
Contribution TO others	3.806	90.223	3.866	82.514	61.622	47.803	85.499	66.48	441.813
Contribution including own	92.534	116.517	90.421	109.833	96.697	85.383	112.125	96.489	TCI
Net spillovers	− 7.466	16.517	− 9.579	9.833	− 3.303	− 14.617	12.125	− 3.511	55.227

Results are based on a TVP-VAR with lag length of order 1 and a 10-step-ahead forecast

**Table 3:** Dynamic connectedness of oil aggregate demand shock

	Aggregate demand shock	FTSE100	DAX30	CAC40	TSX60	FTSE MIB	S&P500	N225	FROM
Aggregate demand shock	83.071	1.761	1.011	3.1	2.828	2.422	2.325	3.482	16.929
FTSE100	0.481	26.161	0.459	18.34	15.42	9.059	17.289	12.791	73.839
DAX30	1.406	1.974	87.047	1.844	3.171	1.308	2.134	1.115	12.953
CAC40	0.257	19.14	0.512	27.294	11.129	8.295	19.457	13.915	72.706
S&P TSX	0.643	20.024	0.316	13.736	34.954	7.37	12.816	10.14	65.046
FTSE MIB	0.432	13.589	0.378	12.022	9.346	37.906	14.421	11.908	62.094
SPY	0.421	17.605	0.257	19.016	10.089	10.168	26.532	15.914	73.468
N225	0.518	15.714	0.258	15.695	10.388	9.441	18.193	29.791	70.209
Contribution TO others	4.158	89.806	3.191	83.752	62.371	48.063	86.635	69.266	447.243
Contribution including own	87.229	115.968	90.238	111.046	97.325	85.968	113.167	99.058	TCI
Net spillovers	− 12.771	15.968	− 9.762	11.046	− 2.675	− 14.032	13.167	− 0.942	55.905

Results are based on a TVP-VAR with lag length of order 1 and a 10-step-ahead forecast

**Table 4:** Dynamic connectedness of oil specific demand shock

	Specific Demand Shock	FTSE100	DAX30	CAC40	TSX60	FTSE MIB	S&P500	N225	FROM
Specific demand shock	78.877	3.233	0.433	4.294	2.33	2.347	3.252	5.233	21.123
FTSE100	0.387	26.174	0.521	18.224	15.543	9.025	17.479	12.648	73.826
DAX30	0.554	2.078	87.417	1.961	3.374	1.363	2.174	1.078	12.583
CAC40	0.647	19.095	0.511	27.366	11.137	8.196	19.398	13.652	72.634
S&P TSX	0.324	20.251	0.337	13.726	35.003	7.402	12.964	9.993	64.997
FTSE MIB	0.985	13.546	0.414	11.811	9.375	37.731	14.234	11.903	62.269
SPY	0.585	17.781	0.27	18.909	10.146	10.091	26.538	15.679	73.462
N225	1.23	15.569	0.241	15.386	10.237	9.509	17.974	29.853	70.147
Contribution TO others	4.713	91.553	2.728	84.311	62.143	47.933	87.475	70.186	451.041
Contribution including own	83.59	117.727	90.145	111.677	97.145	85.664	114.013	100.039	TCI
Net spillovers	− 16.41	17.727	− 9.855	11.677	− 2.855	− 14.336	14.013	0.039	56.38

Results are based on a TVP-VAR with lag length of order 1 and a 10-step-ahead forecast

These graphs show the spillover connectedness effects between G7 stock returns and the three different types of oil price shocks computed via SVAR. The time-varying net directional spillover across eight variables is based on a TVP-VAR method, an extension of the generalized variance decomposition approach (Diebold and Yilmaz 2012).

**Fig. 1:**
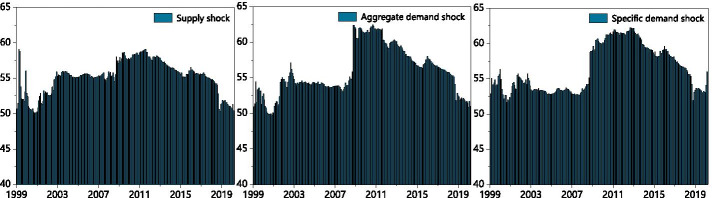
Dynamic total connectedness. *Note*: The total connectedness index is measured with a 10-step ahead forecast horizon and 70-month rolling window

The total impact spillover index during the sample period based on 80-month rolling windows and a 10-step-ahead forecast horizon^2^ is shown in Fig. 1. The three oil price shocks show similar but unidentical fluctuations in the stock return spillover effects of G7 countries. The average of the dynamic connectedness index was estimated at 62.44%. The most significant spike among the three oil shocks was the aggregate demand shock, which fluctuated from 55 to 62% in September of 2008. When international oil prices were hit by the global financial crisis, crude oil jumped from US$ 145.1 (July 6) to US$ 77.7 per barrel after the international market opened (October 5)—a decrease of 46.4%.

Oil supply shock movement spillover was relatively stable after the global financial crisis, but it fluctuated after 2001. This may be attributed to the “9/11” incident which subsequently led to a downturn in aviation, transportation, tourism, and other industries; international oil prices fell from US$ 17 to US$ 16 a barrel during this period. Many countries conducted restorative production to stimulate the economy after the 2008 financial crisis, but this did not significantly affect crude oil supply. The spillover index of oil-specific demand shock substantially increased, ultimately exceeding the spillover effects of supply shock and aggregate demand shock in April of 2002. The connectedness index of the oil supply shock plummeted at the same time.

Global stock markets were also drastically affected by the Wall Street corporate scandal. In March and April of 2002, US, European, and Japanese stocks plummeted as oil was favored by investors as a common hedging commodity. The spillovers of the two oil demand shocks were relatively stable while the total connectedness index of oil supply shocks cyclically fluctuated before 2008. As expected, the connectedness indices of the three oil price shocks spiked due to the intensification of financial crises in August of 2008.

From the last quarter of 2008 to the final month of the sample, the total connectedness indices of the oil shocks also remained higher than previously estimated results. The indices then decreased yearly, preceding a vertiginous drop upon the breakout of the trade conflict between China and the United States. The negative impact that trade protectionism exerts on economic efficiency is new evidence (e.g., by curbing both the supply and demand of crude oil). It is also worth noting that the spillover effect of oil-specific demand shock spread significantly after the COVID-19 outbreak, while that of oil supply shock and oil aggregate demand shock sharply declined. The COVID-19 pandemic appears to have severe implications on the supply of crude oil due to sharp decreases in both productivity and demand (Sharif et al. 2020). The downturn of the stock market encourages investors to move their funds to the crude oil market for hedging purposes.

We next examine the correlations between the decomposed oil shocks and stock returns across different G7 countries by computing the net spillover effects. We explore these dynamic relationships accordingly and determine which variables are the transmitters (receivers) of spillover effects.

**Fig. 2:**
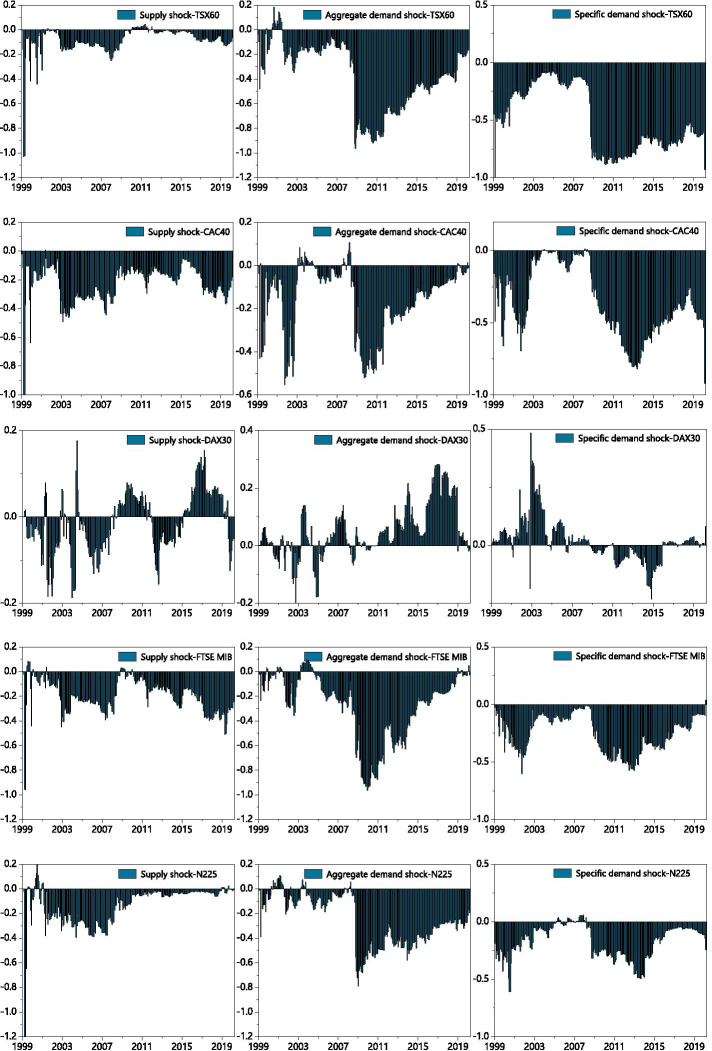

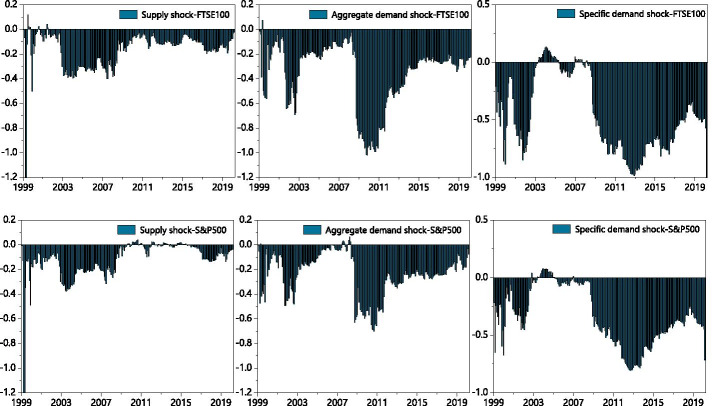
Net pairwise total directional connectedness. *Note*: The net pairwise connectedness index is measured with a 10-step ahead forecast horizon and 70-month rolling window

As shown in Fig. 2, the directional correlations between the stock returns of the seven countries and the decomposed oil price shocks are time-varying and bidirectional in two demand shock types. However, oil supply shock is a net receiver of spillover effects for all G7 member countries, except Germany, within the sample period. This is consistent with the observation of Husain et al. (2019) on the dominant impact of stock returns on oil prices. This effect may be attributable to the transmission mechanism of information mainly proceeding from the stock market to the oil market, and the relatively weak influence of exogenous oil supply on the long-term cash flow of the countries’ primary companies (Nadal et al. 2017).

We also notice that the effects of oil price shocks from different sources on stock returns vary after a disastrous event, especially for net oil importer countries (Mokni 2020). After 9/11, the spillover effects of the two different oil demand prices on the stock returns of G7 countries suddenly increased. Among them, the TSX60, FTSE MIB, and N225 indices first became receivers, then immediately turned into initiators of the spillover effect. The spillover effect of oil supply prices on G7 stock returns spiked post-9/11; subsequent spillover effects decreased to varying extent among the G7 countries. France, Italy, Japan, and the United Kingdom all appear to be sensitive to oil supply price shock.

The 2003 Iraq War also appeared to have stimulated spillover volatility in aggregate and specific demand shocks while the spillover effects of the supply shock gradually decreased. The growth of the global economy was accompanied by a widespread increase in oil consumption. In 2004, the world economic growth rate reached 5%, marking a 30-year peak. Global oil consumption then grew at an average annual rate of 1.8%, reaching one million barrels of oil per day by 2008. Although global demand remains strong, the growth of aggregate oil production by non-OPEC members is currently depressed. Since 2003, global oil consumption has grown faster than annual oil production in non-OPEC members. We also find that events such as the unstable political situation in Venezuela in 2003 and violent conflict in the delta region of Nigeria in 2006 decreased oil supply shocks. The spillover effect of specific oil demands on stock returns in 2005 affected oil exporters (Canada and the United States) and importers (France, Japan, Italy) differently. This may be attributable to the Kyoto Agreement having limited emissions of greenhouse gases, suppressing the demand for oil. The connectedness associated with oil-specific demand shocks was also transmitted to receivers in the United States and the United Kingdom in July of 2005. The largest supply break in US history occurred at this time due to natural disasters having created rampant market anxiety. Other countries showed various degrees of fluctuations in the connectedness of oil-specific demand shocks after the Middle East conflict of 2011; all G7 countries, except Japan, showed a sudden drop in that year.

When global systemic financial crises occur, the spillover in different oil price shocks vary by country due to differences in the dependence on oil imports and exports. This results in a turbulent increase of the spillover effect in terms of oil supply shocks. During the global financial crisis, oil supply shocks on the S&P500 and TSX60 indices increased significantly before dominating the spillover effect. It appears that the crisis had a greater impact on countries with larger oil export volumes, which is in accordance with the previous observations of Mokni (2020).

The net transmission of volatility spillover values towards aggregate demand shock dropped significantly during the financial crisis as well. There was abundant oil supply and weak oil demand at that time, which drove the bulls to flee and encouraged short-selling in traders. In effect: oil prices are determined by the demand side. Fantazzini (2016) similarly found a negative bubble as oil prices plunged during this period. Additionally, after the Middle East conflict of 2011, a reduction in oil output pushed the spillover of aggregate demand shocks to increase annually through the end of our sample period.

Compared with the oil supply price shock and oil aggregate demand price shock, the spillover effect of oil-specific demand shock on G7 stock returns is worth more concern. The COVID-19 outbreak, for example, has markedly decreased the spillover effect of G7 countries, except for Germany and Italy. In fact, the influence of stock returns has dramatically expanded to an even greater extent than in 2008. The COVID-19 outbreak has caused the supply of and demand for oil to widely diverge from investors’ expectations on oil and normal stock market conditions. Volatility in the stock market also determines the speculating price of oil, that is, there is an asymmetrical shift of risk from the oil market to the stock market (Maghyereh et al. 2016).

Oil price is a systematic risk variable that affects stock returns (Thorbecke 2019). Our TVP-VAR results are in line with Nadal’s et al. (2017), where oil demand shocks deeply affect the relationships at work throughout the sample period. The stock market environment of developed G7 economies also has a certain impact on oil price volatility. Our spillover observations also reveal that the relationship between oil prices and stock returns is deeply affected by the risk of an extreme tail event. The relationships between oil and stock prices at the distributional level thus merit careful analysis.

### Quantile-on-quantile

**Fig. 3:**
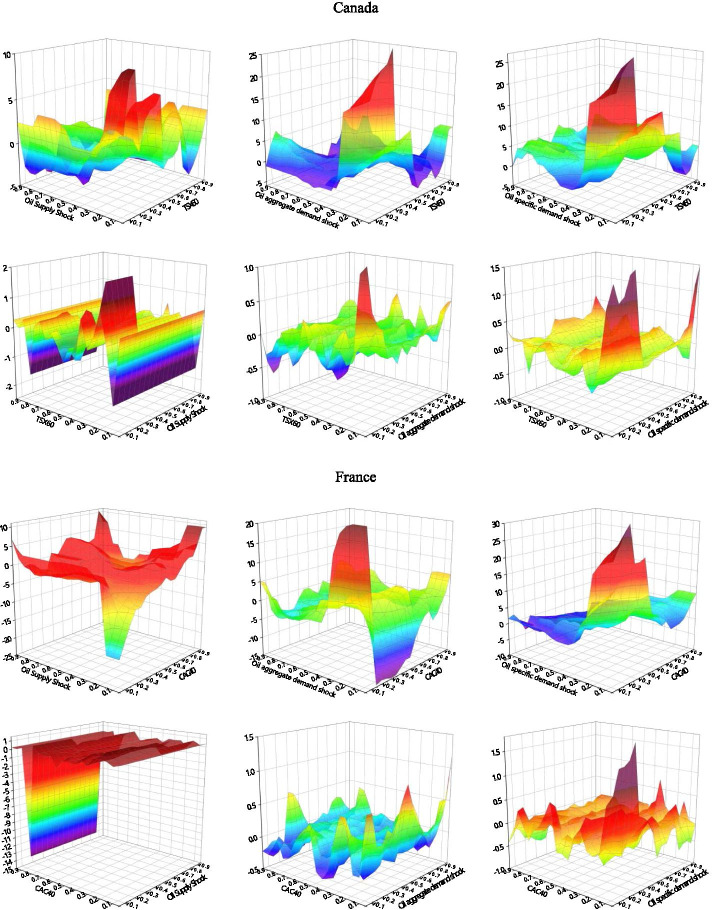

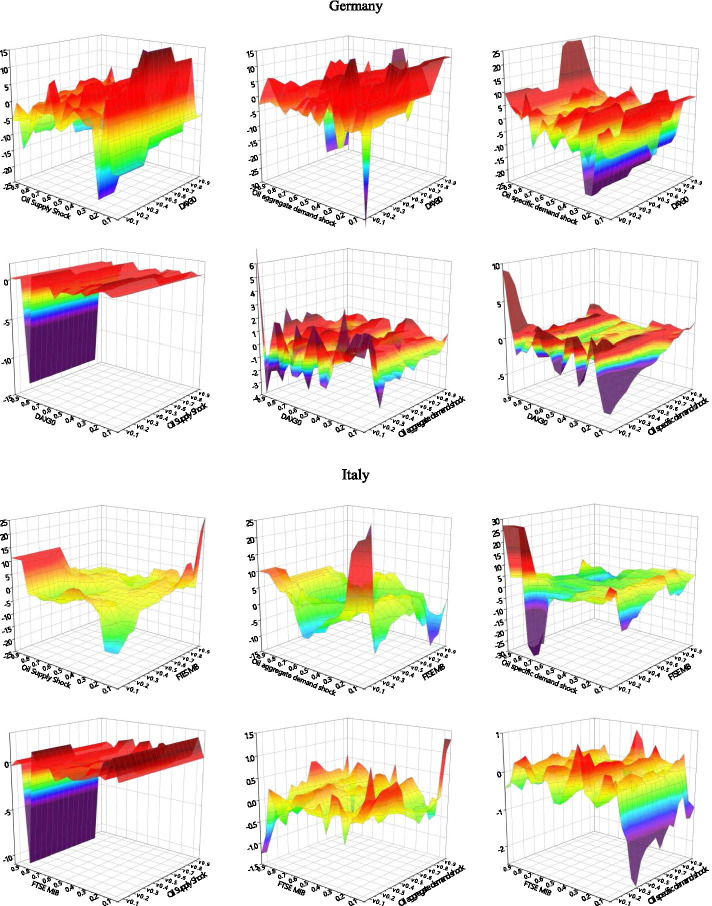

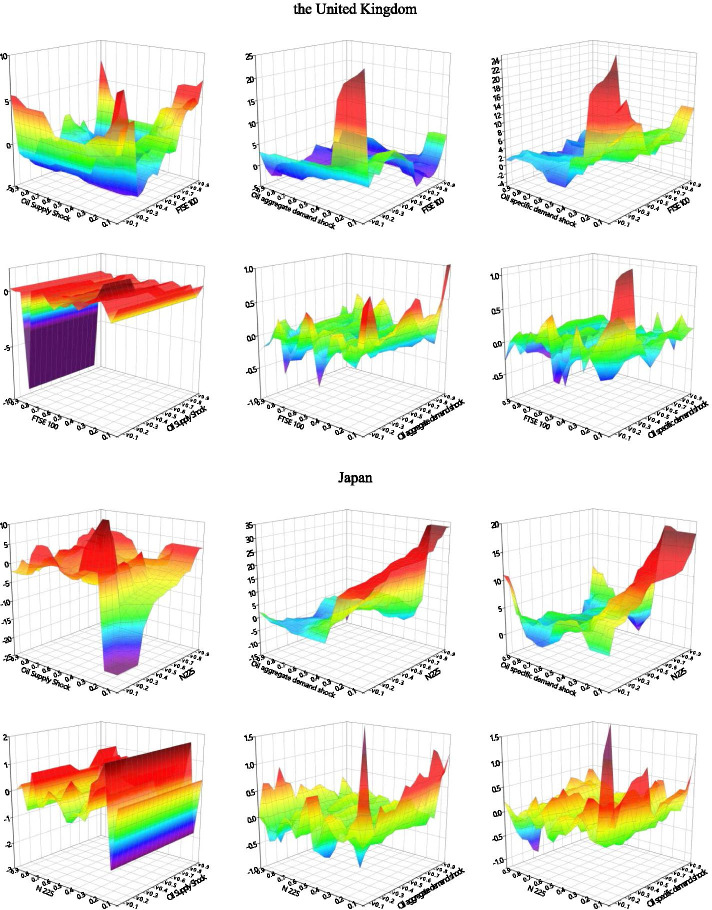

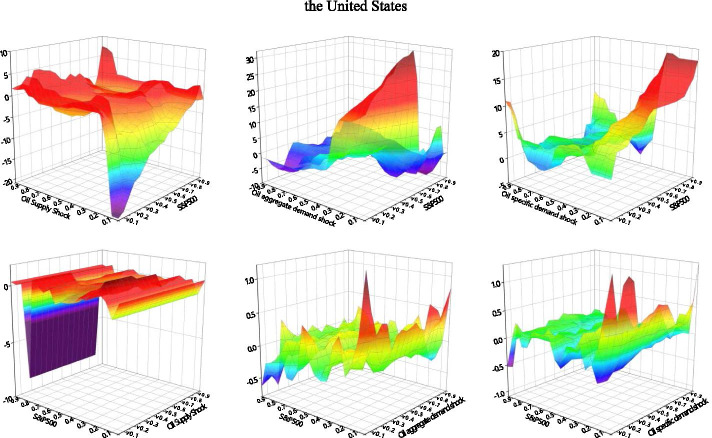
Correlation between decomposed oil shocks and stock returns applying the QQ approach. *Note*: The graphs illustrate the results of the slope coefficient, $\beta_{1} (\theta ,\tau )$ situated on the z-axis against the quantiles of decomposed oil shocks ($\theta$) on the x-axis and the quantiles of the stock returns ($\tau$) on the y-axis. The first line of each country is the stock markets impact the oil shocks, and the second line of each country is the effect of oil shocks on stock returns

We use the QQ method to analyze G7 stock return information in response to different types of decomposed oil price shocks. We explore their impact structures under different stock market shocks, as well as the detailed effects of different quantiles upon impact. As shown in Fig. 3, the effects between decomposed oil price shocks and stock returns across different stock market conditions are not uniform.

The effects of oil supply shocks on stock returns can be divided into three categories. The first involves oil-importers (France, Germany, Italy, the United Kingdom, the United States), where we observe considerable asymmetrical effects, including soft undulated effects, at the low quantiles (0.05–0.30) and strong negative effects at the high quantiles (0.70–0.85) of stock returns. This is in line with the observations of Sadorsky (1999), Lee and Chiou (2011), Reboredo and Ugolini (2016), and Ewing et al. (2018), where the correlations of high stock returns to the negative oil supply shock can be positive. The second category involves the net oil-exporting country (Canada), which shows intensive negative effects at both the low and high quantiles (0.10–0.25, 0.70–0.85) of stock returns. The third category involves the net oil importer in Asia (Japan), which exhibits negative effects at the low quantiles of stock returns. The oil supply price stimulates significant fluctuations in stock returns when stock prices are extremely low. In this case, a normal oil supply shock would more effectively promote economic recovery than a low oil supply shock. Therefore, oil-importing countries may be more interested in normal than lower oil prices under declining stock price conditions. This negative impact on the depressed stock market is particularly acute in resource-poor net oil importers (Japan).

We also find that the effects of stock returns on oil supply shocks are relatively stable when the stock market environment is smooth. Intense negative effects are observed at the lower quantiles (0.05–0.25) of oil supply shock in most countries, while an asymmetrical effect emerged at the low and high quantiles of the TSX60 in Canada. Oil supply shocks at low quantiles (0.05–0.70) appear to have a positive effect on stock returns, while high quantiles (0.70–0.95) of oil supply shocks have a significant negative effect. The significant positive effect observed in Canada in the low quantiles of oil supply shock and stock returns may be due to the fact that it is a net oil-exporting country, where rigid demand for oil and high oil prices characterize an active domestic market (Wang et al. 2013). Except for Canada and Germany, which show a negative impact in the very high quantile area of stock returns, all G7 countries show an asymmetrical impact at the high quantiles of oil shocks. As we expected, an increase in oil prices was followed by slower output growth (Hamilton 1983).

We mainly observe positive correlations at the low quantiles of oil aggregate demand shocks with low quantiles of stock returns in most countries—Japan shows a somewhat positive relationship at the low quantiles (0.05–0.95) of stock returns with the whole quantiles of oil shocks. We also find asymmetrical effects in the quantiles of demand shocks on those of stock returns within the extreme quantiles of the two respective variables. The stock prices of developed countries appear to be stimulated by normal and exceptionally high oil demands. In most of the G7 countries, this effect intensified—and grew increasingly complex—as stock prices increased. These findings are similar to those of Lee and Zeng. (2011), Gong and Lin (2018), and Mokni (2020).

With regard to changes in stock returns derived from the oil aggregate demand shock, we find positive effects in the Italian, the United Kingdom, and the United States low quantiles of oil aggregate demand shocks in the lower quantiles of stock returns. We observe intense negative effects at the low quantiles of oil shock with low quantiles (0.15–0.20) of stock returns and positive effects at relatively high quantiles (0.70–0.85) of stock returns in all the G7 countries. Japan shows a stronger positive relationship at the low quantiles of oil demand prices, and aggregate-demand prices were more sensitive to stock price fluctuations in European countries (France, Germany, Italy) than others. Developed economies with booming stock markets appear to have greater oil aggregate demand; the asymmetrical relationship when demand for oil was low suggests that higher anomalous returns were driven by rising oil prices, which is an evidence of economic growth (Brook et al. 2004).

The effects of oil-specific demand shocks on stock returns are similar to the effect of oil demand prices on stock returns; asymmetrical effects were found at the lower (0.05–0.0.30) and higher (0.70–0.95) quantiles of stock returns in most G7 countries. The positive effects gradually change into negative effects as the quantile of stock returns increased to reach the bottom of the high quantile. Germany is the only exception to this rule, as its volatility increased as stock return quantiles rose. Oil price shocks show positive effects on stock markets, peaking at the low-to-middle oil price quantiles (0.25–0.40).

The above results suggest that oil price volatility significantly influences investor sensitivity across different asset markets. The effects of low oil-specific demand shocks differ within different stock market environments (Mokni 2020). When more confident investors navigate the market, high oil prices have more positive effects on stock prices than a market dominated by less-confident investors. When oil prices and the market are relatively stable, this effect becomes particularly intense. For most G7 countries, high oil prices appear to have a positive impact on (stable) stock markets.

Within the responses of oil-specific demand shocks to stock returns, we observe intensely fluctuating correlations between low stock return quantiles (0.05–0.30) with low-to-medium oil shock quantiles (0.05–0.40). Surprisingly, the sharp effects alternated between the positive and negative at the low (0.05–0.30) and high (0.70–0.90) quantiles of stock returns with high oil shock quantiles. These results imply that investors in G7 markets are markedly influenced by stock volatility; they tend to show strong investment willingness in the initial recovery of the stock market and in bull markets.

High oil prices may exert asymmetrical effects on G7 stock markets (Lee and Zeng 2011; Gong and Lin 2018; Mokni 2020). The spillover effects of oil shocks dramatically fluctuate in the wake of plummeting stock prices (such as in 2008 and 2020) due to the negative impact of extremely low stock returns on higher oil prices. Our QQ method analysis confirms this impact of oil shocks on stocks at lower levels. Oil-importing countries such as Japan, that rely heavily on oil for economic development, are more sensitive to such extreme risks. Conversely, the stock markets of oil-exporters like Canada and the United States, are more likely to be affected by elevated oil prices when equity markets perform poorly. Our QQ results show that in addition to the tail effects, the effects of oil supply shocks on stock returns are significantly greater than those of stock returns on oil supply shocks. The oil supply shock dominates the spillover.

## Conclusion

In this study, we utilize data from G7 countries from January 1999 to March 2020 to investigate the effects of oil shocks on stock returns under the condition of oil decomposition. We attempt to capture the overall dynamic connectedness of stock return-dependent spillover on decomposed oil shocks across the whole sample period. We also investigate the impact structure between different quantiles of decomposed oil shocks and stock returns under various stock market conditions. Our main findings can be summarized as follows.

The TVP-VAR method we use to observe volatility co-movements reveals a time-varying relationship between the decomposed oil price shocks and stock returns of each G7 country. Oil price shock spillover values affect stock returns differently from varying sources. Aggregate and specific demand shocks transmit more spillovers to stock returns under different stock market conditions, while supply shock receives more spillovers from each country. The oil supply shock is a net receiver of spillover effects for all G7 member countries within the sample period; for most of these countries, oil supply shock fluctuations are affected by share returns. Oil demand shock is more impactful immediately upon the breakout of a financial crisis. Filis et al. (2011) and Ahmadi et al. (2016) reached similar conclusions. It is crucial to effectively manage oil demand fluctuations during any global crisis. The directional spillover risk between oil and the stock markets during the COVID-19 pandemic and the global financial crisis of 2008 are stark examples of this. The aggregate demand of oil was intensely affected by the crisis in 2008, though the outbreak was slightly delayed. The COVID-19 pandemic has exerted the greatest spillover effect on the specific demand for oil, and has engulfed the world’s economy at a tremendous speed.

We employ a QQ model to analyze the above effects. We find no general correlation between decomposed oil shocks and stock returns in any G7 country. The QQ approach effectively demonstrates the reaction of different quantiles of stock returns under the impact of the same quantile of oil price. Oil price changes are explained here using general financial system information. Oil price shocks from different sources do not show different co-movements with G7 stock returns. In previous studies, high oil prices showed high QQ coefficients with stock returns. Our empirical results reveal more details at the distributional level. We find that the impact of the same source of oil prices on stock returns across different stock market conditions is heterogeneous.

On one hand, oil supply shock generally depresses stock prices. Stock returns in the net oil-exporting country (Canada) are more significantly affected by oil price shock compared to other countries. On the other hand, impact on oil-importing countries in Europe display a light positive effect during the low to middle quantiles of stock return. The relationships in the oil aggregate shock and specific demand shock are more intensively affected by oil price volatility and stock fluctuations compared to oil supply shock (Bastianin et al. 2016). Furthermore, in each country, significant variations can be observed between three types of oil shocks and stock returns at different quantiles. This indicates that the distribution among the variables is not uniform across quantiles and is related to the country’s dependence on oil imports and exports. We also find that there are considerable asymmetric effects at the extreme tails, with low quantiles of oil price shock showing the most significant performance. These conclusions are consistent with the results of Sadorsky (1999), Lee and Chiou (2011), and Reboredo and Ugolini (2016), who observed asymmetrical effects in upward and downward oil price volatility on stock markets.

The positive impact of steady stock and oil markets on economic operation stability, and the heterogenous adverse effects between decomposed oil prices and G7 stock returns, have the following implications:

Firstly, policymakers and shareholders must calm markets and establish investments, in addition to being aware of external risk spillovers and paying attention to tension tracking and observation mechanism. Doing so can help minimize possible risks of spillovers triggered by the fear effect arising from investors’ herding behavior. Second, according to different oil shocks, oil-importing countries such as Japan should not only guard against the systemic risks of higher oil prices, but also pay more attention to stock conditions, especially in bear markets. This can prevent stock markets from suffering downturn risks when the demand price of oil plummets. Oil exporters such as Canada, should take into account both low and high share prices. Third, due to the complex and sensitive relationship between special demand prices and the stock market, investors in global financial markets, global risk managers, and stakeholders should pay more attention to the volatility of the stock market. Moreover, to reduce information asymmetry and the risk of stock market crashes, they must beware of systemic risks while maximizing their investment portfolio (Wen et al. 2019), especially during the COVID-19 pandemic. With regard to price changes on the oil supply side, investors in countries like Canada and Japan may have greater opportunities.

Focusing on the co-movement between the decomposition oil price and the stock markets of G7, our study reveals various stock markets respond heterogeneously to different oil prices. And we further draw similarities and differences between the risk contagion in 2020 and 2008. Nevertheless, we haven’t taken into account the factors which may affect the stock market such as the number of infections, the economic policy uncertainty as well as geopolitical risks just as Sharif et al. (2020) did. The future research will include the aforementioned elements to gain a clearer insight into the correlations between the oil price market and the stock market.

## Footnotes

1 The data is available at https://finance.yahoo.com/.

2 The size of the rolling window was set around 1/3 of the observations (254). We have also investigated the robustness of our results based on alternative rolling windows from a 30- to 150-month and a forecast horizon from 5- to 15-months. The results are qualitatively very similar.

## References

[CR1] Ahmadi M, Manera M, Sadeghzadeh M (2016). Global oil market and the US stock returns. Energy.

[CR2] Aloui R, Hammoudeh S, Nguyen DK (2013). A time-varying copula approach to oil and stock market dependence: the case of transition economies. Energy Econ.

[CR3] Antonakakis N, Gabauer D (2017) Refined measures of dynamic connectedness based on TVP-VAR. MPRA Paper 78282, University Library of Munich, Germany

[CR4] Antonakakis N, Chatziantoniou I, Filis G (2017). Oil shocks and stock markets: dynamic connectedness under the prism of recent geopolitical and economic unrest. Int Rev Financ Anal.

[CR5] Antonakakis N, Cunado J, Filis G, Gabauer D, De Gracia FP (2018). Oil volatility, oil and gas firms and portfolio diversification. Energy Econ.

[CR6] Balassa B (1985). Exports, policy choices, and economic growth in developing countries after the 1973 oil shock. J Dev Econ.

[CR7] Basher SA, Haug AA, Sadorsky P (2012). Oil prices, stock returns and emerging stock markets. Energy Econ.

[CR8] Bastianin A, Conti F, Manera M (2016). The impacts of oil price shocks on stock market volatility: evidence from the G7 countries. Energy Policy.

[CR9] Bjørnland HC (2009). Oil price shocks and stock market booms in an oil exporting country. Scott J Polit Econ.

[CR10] Bouoiyour J, Selmi R (2016) The infernal couple China-Oil Price and the Responses of G7 equities: a QQ approach. MPRA Paper 70379, University Library of Munich, Germany

[CR11] Brook AM, Price R, Sutherland D, Westerlund N, André C (2004) Oil price developments: drivers, economic consequences and policy responses. OECD Economics Working Paper No. 412, December 8

[CR12] Chao X, Kou G, Peng Y, Viedma EH (2020). Large-scale group decision-making with non-cooperative behaviors and heterogeneous preferences: an application in financial inclusion. Eur J Oper Res.

[CR13] Chen SS (2014). Forecasting crude oil price movements with oil-sensitive stocks. Econ Inq.

[CR14] Chang BH, Sharif A, Aman A, Suki NM, Salman A, Khan SAR (2020). The asymmetric effects of oil price on sectoral Islamic stocks: new evidence from quantile-on-quantile regression approach. Resour Policy.

[CR15] Cuñado J, De Gracia FP (2003). Do oil price shocks matter? Evidence for some European countries. Energy Econ.

[CR16] Cuñado J, De Gracia FP (2005). Oil prices, economic activity and inflation: evidence for some Asian countries. Q Rev Econ Financ.

[CR17] Cuñado J, Jo S, de Gracia FP (2015). Macroeconomic impacts of oil price shocks in Asian economies. Energy Policy.

[CR18] Diaz EM, Molero JC, de Gracia FP (2016). Oil price volatility and stock returns in the G7 economies. Energy Econ.

[CR19] Diebold FX, Yilmaz K (2009). Measuring financial asset return and volatility spillovers, with application to global equity markets. Econ J.

[CR20] Diebold FX, Yilmaz K (2012). Better to give than to receive: predictive directional measurement of volatility spillovers. Int J Forecast.

[CR21] Diebold FX, Liu L, Yilmaz K (2017) Commodity connectedness (No. w23685). National Bureau of Economic Research

[CR22] Du L, Yanan H, Wei C (2010). The relationship between oil price shocks and China’s macro-economy: an empirical analysis. Energy Policy.

[CR23] Ewing BT, Kang W, Ratti RA (2018). The dynamic effects of oil supply shocks on the US stock market returns of upstream oil and gas companies. Energy Econ.

[CR24] Fang CR, You SY (2014). The impact of oil price shocks on the large emerging countries' stock prices: evidence from China, India and Russia. Int Rev Econ Financ.

[CR25] Fantazzini D (2016). The oil price crash in 2014/15: was there a (negative) financial bubble?. Energy Policy.

[CR26] Ferrer R, Shahzad SJH, López R, Jareño F (2018). Time and frequency dynamics of connectedness between renewable energy stocks and crude oil prices. Energy Econ.

[CR27] Filis G, Degiannakis S, Floros C (2011). Dynamic correlation between stock market and oil prices: the case of oil-importing and oil-exporting countries. Int Rev Financ Anal.

[CR28] Gong X, Lin B (2018). Time-varying effects of oil supply and demand shocks on China's macro-economy. Energy.

[CR29] Hamilton JD (1983). Oil and the macroeconomy since World War II. J Polit Econ.

[CR30] Hamilton JD, Durlauf S, Blume L (2008). Oil and the macroeconomy. The new palgrave dictionary of economics.

[CR31] Husain S, Tiwari AK, Sohag K, Shahbaz M (2019). Connectedness among crude oil prices, stock index and metal prices: an application of network approach in the USA. Resour Policy.

[CR33] Ji Q, Liu ML, Fan Y (2015). Effects of structural oil shocks on output, exchange rate, and inflation in the BRICK countries: a structural vector autoregression approach. Emerg Mark Financ Trade.

[CR32] Ji Q, Geng JB, Tiwari AK (2018). Information spillovers and connectedness networks in the oil and gas markets. Energy Econ.

[CR35] Jiang Y, Lao J, Mo B, Nie H (2018). Dynamic linkages among global oil market, agricultural raw material markets and metal markets: an application of wavelet and copula approaches. Phys A Stat Mech Appl.

[CR34] Jiang Y, Feng Q, Mo B, Nie H (2020). Visiting the effects of oil price shocks on exchange rates: quantile-on-quantile and causality-in-quantiles approaches. N Am J Econ Financ.

[CR36] Jones CM, Kaul G (1996). Oil and the stock markets. J Financ.

[CR37] Kang W, Ratti RA (2013). Oil shocks, policy uncertainty and stock market return. J Int Financ Mark Inst Money.

[CR38] Kaul G, Seyhun HN (1990). Relative price variability, real shocks, and the stock market. J Financ.

[CR39] Kilian L (2009). Not all oil price shocks are alike: disentangling demand and supply shocks in the crude oil market. Am Econ Rev.

[CR40] Kilian L, Park C (2009). The impact of oil price shocks on the US stock market. Int Econ Rev.

[CR41] Koenker R, Bassett G (1978). Regression quantiles. Econom J Econom Soc.

[CR42] Koop G, Pesaran MH, Potter SM (1996). Impulse response analysis in nonlinear multivariate models. J Econom.

[CR43] Kou G, Peng Y, Wang G (2014). Evaluation of clustering algorithms for financial risk analysis using MCDM methods. Inf Sci.

[CR46] Lee YH, Chiou JS (2011). Oil sensitivity and its asymmetric impact on the stock market. Energy.

[CR44] Lee CC, Zeng JH (2011). The impact of oil price shocks on stock market activities: asymmetric effect with quantile regression. Math Comput Simul.

[CR45] Lee K, Ni S, Ratti RA (1995). Oil shocks and the macroeconomy: the role of price variability. Energy J.

[CR47] Leung GC (2010). China's oil use, 1990–2008. Energy Policy.

[CR48] Li Q, Cheng K, Yang X (2017). Response pattern of stock returns to international oil price shocks: from the perspective of China’s oil industrial chain. Appl Energy.

[CR49] Lin B, Su T (2020). The linkages between oil market uncertainty and Islamic stock markets: evidence from quantile-on-quantile approach. Energy Econ.

[CR50] Ma L, Koenker R (2006). Quantile regression methods for recursive structural equation models. J Econom.

[CR51] Maghyereh AI, Awartani B, Bouri E (2016). The directional volatility connectedness between crude oil and equity markets: new evidence from implied volatility indexes. Energy Econ.

[CR52] Mensi W, Hammoudeh S, Reboredo JC, Nguyen DK (2014). Do global factors impact G7 stock markets? A quantile regression approach. Emerg Mark Rev.

[CR53] Morana C (2013). Oil price dynamics, macro-finance interactions and the role of financial speculation. J Bank Financ.

[CR54] Mork KA (1989). Oil and the macroeconomy when prices go up and down: an extension of Hamilton's results. J Polit Econ.

[CR55] Mokni K (2020). Time-varying effect of oil price shocks on the stock market returns: evidence from oil-importing and oil-exporting countries. Energy Rep.

[CR56] Nadal R, Szklo A, Lucena A (2017). Time-varying impacts of demand and supply oil shocks on correlations between crude oil prices and stock markets indices. Res Int Bus Financ.

[CR57] Narayan PK, Narayan S (2007). Modelling oil price volatility. Energy Policy.

[CR58] Oladosu GA, Leiby PN, Bowman DC, Uría-Martínez R, Johnson MM (2018). Impacts of oil price shocks on the United States economy: a meta-analysis of the oil price elasticity of GDP for net oil-importing economies. Energy Policy.

[CR59] Papapetrou E (2001). Oil price shocks, stock market, economic activity and employment in Greece. Energy Econ.

[CR60] Park J, Ratti RA (2008). Oil price shocks and stock markets in the US and 13 European countries. Energy Econ.

[CR61] Pesaran MH, Shin Y (1998). An autoregressive distributed-lag modelling approach to cointegration analysis. Econom Soc Monogr.

[CR62] Phan DHB, Tran VT, Nguyen DT (2019). Crude oil price uncertainty and corporate investment: new global evidence. Energy Econ.

[CR63] Reboredo JC (2015). Is there dependence and systemic risk between oil and renewable energy stock prices?. Energy Econ.

[CR64] Reboredo JC, Rivera-Castro MA (2014). Wavelet-based evidence of the impact of oil prices on stock returns. Int Rev Econ Financ.

[CR65] Reboredo JC, Ugolini A (2016). Quantile dependence of oil price movements and stock returns. Energy Econ.

[CR66] Sadorsky P (1999). Oil price shocks and stock market activity. Energy Econ.

[CR67] Shahbaz M, Zakaria M, Shahzad SJH, Mahalik MK (2018). The energy consumption and economic growth nexus in top ten energy-consuming countries: fresh evidence from using the quantile-on-quantile approach. Energy Econ.

[CR68] Sharif A, Afshan S, Qureshi MA (2019). Idolization and ramification between globalization and ecological footprints: evidence from quantile-on-quantile approach. Environ Sci Pollut Res.

[CR69] Sharif A, Aloui C, Yarovaya L (2020). COVID-19 pandemic, oil prices, stock market, geopolitical risk and policy uncertainty nexus in the US economy: fresh evidence from the wavelet-based approach. Int Rev Financ Anal.

[CR70] Sim N, Zhou H (2015). Oil prices, US stock return, and the dependence between their quantiles. J Bank Financ.

[CR71] Smyth R, Narayan PK (2018). What do we know about oil prices and stock returns?. Int Rev Financ Anal.

[CR72] Taghizadeh-Hesary F, Yoshino N, Mohammadi Hossein Abadi M, Farboudmanesh R (2016). Response of macro variables of emerging and developed oil importers to oil price movements. J Asia Pac Econ.

[CR73] Thorbecke W (2019). Oil prices and the US economy: evidence from the stock market. J Macroecon.

[CR74] Wang Y, Wu C, Yang L (2013). Oil price shocks and stock market activities: evidence from oil-importing and oil-exporting countries. J Comp Econ.

[CR75] Wang Y, Wu C, Yang L (2014). Oil price shocks and agricultural commodity prices. Energy Econ.

[CR76] Wang H, Kou G, Peng Y (2020). Multi-class misclassification cost matrix for credit ratings in peer-to-peer lending. J Oper Res Soc.

[CR77] Wen F, Xu L, Ouyang G, Kou G (2019). Retail investor attention and stock price crash risk: evidence from China. Int Rev Financ Anal.

[CR79] Zhang YJ (2013). Speculative trading and WTI crude oil futures price movement: an empirical analysis. Appl Energy.

[CR78] Zhang D (2017). Oil shocks and stock markets revisited: measuring connectedness from a global perspective. Energy Econ.

[CR80] Zhu H, Guo Y, You W, Xu Y (2016). The heterogeneity dependence between crude oil price changes and industry stock market returns in China: evidence from a quantile regression approach. Energy Econ.

